# Introducing and Validating the Multiphasic Evidential Decision-Making Matrix (MedMax) for Clinical Management in Patients with Intrahepatic Cholangiocarcinoma

**DOI:** 10.3390/cancers17010052

**Published:** 2024-12-27

**Authors:** Ali Ramouz, Ali Adeliansedehi, Elias Khajeh, Keno März, Dominik Michael, Martin Wagner, Beat Peter Müller-Stich, Arianeb Mehrabi, Ali Majlesara

**Affiliations:** 1 Department of General, Visceral and Transplantation Surgery, University of Heidelberg, 69120 Heidelberg, Germany; 2 National Center for Tumor Diseases (NCT) Heidelberg, 69120 Heidelberg, Germany; 3 Liver Cancer Center Heidelberg (LCCH), University of Heidelberg, 69120 Heidelberg, Germany; 4 Division of Computer Assisted Medical Interventions (CAMI), German Cancer Research Center (DKFZ), 69120 Heidelberg, Germany; 5 Center for the Tactile Internet with Human in the Loop (CeTI), Technical University Dresden, 01069 Dresden, Germany; 6 Department of Surgery, Clarunis University Center for Gastrointestinal and Liver Disease, University Hospital and St. Clara Hospital Basel, 4052 Basel, Switzerland

**Keywords:** HPB surgery, cholangiocarcinoma, liver resection, decision making

## Abstract

**Background:** Despite the significant advancements of liver surgery in the last few decades, the survival rate of patients with liver and pancreatic cancers has improved by only 10% in 30 years. Precision medicine offers a patient-centered approach, which, when combined with machine learning, could enhance decision making and treatment outcomes in surgical management of ihCC. This study aims to develop a decision support model to optimize treatment strategies for patients with ihCC, a prevalent primary liver cancer. **Methods:** The decision support model, named MedMax, was developed using three data sources: studies retrieved through a systematic literature review, expert opinions from HPB surgeons, and data from ihCC patients treated at Heidelberg University Hospital. Expert opinions were collected via surveys, with factors rated on a Likert scale, while patient data were used to validate the model’s accuracy. **Results:** The model is structured into four decision-making phases, assessing diagnosis, treatment modality, surgical approach, and prognosis. Prospectively, 44 patients with ihCC were included for internal primary validation of the model. MedMax could predict the appropriate treatment considering the resectability of the lesions in 100% of patients. Also, MedMax could predict a decent surgical approach in 77% of the patients. The model proved effective in making decisions regarding surgery and patient management, demonstrating its potential as a clinical decision support tool. **Conclusions:** MedMax offers a transparent, personalized approach to decision making in HPB surgery, particularly for ihCC patients. Initial results show high accuracy in treatment selection, and the model’s flexibility allows for future expansion to other liver tumors and HPB surgeries. Further validation with larger patient cohorts is required to enhance its clinical utility.

## Simple Summary

Hepatopancreaticobiliary (HPB) surgery has made strides in recent years, yet survival rates for liver cancers have only improved by 10% over 30 years. This study presents a multiphasic evidential decision-making matrix (MedMax), a decision support model developed to optimize treatment for intrahepatic cholangiocarcinoma (ihCC), a primary liver cancer. The model was built using data from randomized controlled trials and expert opinions from HPB surgeons treated at Heidelberg University Hospital. MedMax operates in four phases: diagnosis, treatment modality, surgical approach, and prognosis. It demonstrated high accuracy in supporting surgical and treatment decisions, showing potential as a clinical tool. Further validation is needed with larger patient groups to confirm its broader clinical application.

## 1. Introduction

In recent decades, extensive study and technical innovations in hepatobiliary surgery have greatly advanced the field. These developments have significantly improved diagnosis, surgical approaches, and postoperative care in patients with primary liver or pancreatic cancers [1]. However, survival in this patient population has increased by only 10% in the last 30 years [2,3,4], suggesting that therapeutic approaches and patient management need to be reconsidered. Precision medicine considers individual patient variability, and adapting this approach to the treatment of patients undergoing liver surgery would enable a more tailored, patient-based management strategy [5,6].

Meaningful clinical outcomes are best derived from patient data (e.g., databases), scientific literature, and expert opinions [7,8]. However, it is difficult to extract meaningful data from the vast amount of information in patient databases and the scientific literature [9,10]. Therefore, novel techniques and methodologies are needed, such as machine learning, to use these resources to improve surgical outcomes. Machine learning and data science have made “big data” utilization more accessible and cost-effective and might be able to make individual treatment decisions by evaluating data from patient databases, scientific literature, and expert opinion [11,12,13].

In this study, we are going to design a decision-making matrix to improve treatment and management decisions in patients undergoing liver surgery. Patients with intrahepatic cholangiocarcinoma (ihCC) were considered suitable candidates for establishing a decision support platform because these patients are mainly managed with surgical approaches that expand to all other indications of liver surgeries. Choosing the best treatment modality is important in patients with ihCC because it determines the prognosis. To improve treatment selection and ensure the best possible outcomes in patients with ihCC, we developed a decision support model based on data from clinical databases, scientific literature, and expert opinion.

## 2. Materials and Methods

### 2.1. Materials

The computational model of multiphasic evidential decision-making matrix (MedMax) was based on data from a literature review of all published studies as well as randomized controlled trials (RCT), opinions of experts with at least 15 years of experience in hepatopancreatobiliary (HPB) surgery, and data from patients who underwent surgical treatment for ihCC at our center.

#### 2.1.1. Factor Extraction from the Scientific Literature

A systematic literature review in Medline (via PubMed), Cochrane CENTRAL, and the ISI Web of Science was conducted to identify studies evaluating factors associated with treatment selection, surgical decision making, and postoperative outcomes in patients with ihCC [14,15]. The search included relevant keywords combined with the logical Boolean operators ‘OR’ and ‘AND’. The PubMed search term was as follows:

(liver[tiab] OR hepatic[tiab] OR hepatob*[tiab] OR hepatoc*[tiab] OR biliary[tiab] OR bilio*[tiab] OR gall-bladder[tiab] OR gallbladder[tiab] OR cholecystitis[tiab] OR cholecystolithiasis[tiab] OR choledochal[tiab] OR cholangiocarcinoma[tiab] OR bile-duct[tiab] OR angiosarcoma[tiab] OR hemangioma[tiab] OR focal-nodular-hyperplasia[tiab]) AND (resection* [tiab] OR removal[tiab] OR surger*[tiab] OR surgical[tiab] OR laparotom*[tiab] OR laparoscop*[tiab] OR endoscop*[tiab] OR operation*[tiab] OR operated[tiab] OR surgical-procedures[tiab] OR operative[tiab] OR general-surgery[tiab] OR segmentectomy[tiab] OR bisegmentectomy[tiab] OR trisegmentectomy[tiab] OR sectorectomy[tiab] OR sectionectomy[tiab] OR hepatectomy[tiab] OR lobectomy[tiab] OR enucleation*[tiab] OR deroofing[tiab] OR whipple[tiab] OR cholecystectom*[tiab] OR chemoembolization*[tiab] OR microwave-ablation[tiab] OR radiofrequency-ablation[tiab] OR radiofrequency-treatment[tiab] OR embolectomy[tiab] OR endoscopic-retrograde-cholangiopancreatographic[tiab] OR ERCP[tiab]) AND (randomized controlled trial[pt] OR random*[tw] OR RCT[tw] OR (randomized controlled trials as Topic[MeSH terms]) OR (controlled clinical trial[pt]).

We formulated a study question based on the Population, Intervention, Comparison, Outcome, and Study design. All studies encompassing relevant factors and preoperative data with information on the prediction of intra- and postoperative outcomes and prognosis in patients with ihCC were included. The full articles of interest were then reviewed, and suitable and significant factors were selected for extraction. Extracted preoperative factors included baseline and demographic characteristics, medical history, and diagnostic workups such as laboratory, imaging, and histopathological examinations.

#### 2.1.2. Expert Opinion

After the data and characteristics associated with choosing the treatment in patients with ihCC were retrieved, the potential indicators were formatted into a survey. Twenty-five hepatobiliary surgeons on the tumor board panel were asked to score the association of each factor with the management and postoperative outcomes of patients with ihCC according to the following five-point Likert scale: (1) do not include, (2) little reason to include, (3) could include, (4) should include, and (5) must include. The experts were also invited to comment further and to suggest other influencing factors that were not in the questionnaire. These results were tabulated in an Excel worksheet and the frequencies of the scores for each factor were calculated. All potential elements with an average rating of less than four were excluded from the model design.

#### 2.1.3. Patient Data

To validate the accuracy and feasibility of the model, a prospective collective was obtained from the data of patients with primary liver cancers who were discussed at interdisciplinary liver disease tumor boards between January 2021 and December 2021.The data of adult patients with ihCC (*n* = 44) who had not received emergency surgery were obtained from a prospectively collected database. The collection of patients’ data was approved by the Heidelberg University Hospital ethics committees (approval number: S-754/2018). We excluded all patients with a diagnosis other than ihCC on postoperative histology, with hepatic recurrence of ihCC, who underwent primary R0 resection at Heidelberg University Hospital, who underwent their first surgery at an external hospital and did not have full access to their information and who were diagnosed with perihilar cholangiocellular carcinoma (phCC) and secondary liver involvement. We also excluded patients with missing data on the variables used to create the matrix. In the end, 44 patients were included in the final analyses. Once the factors for model design were determined, the necessary data were extracted for each patient.

Decision making was simulated via MedMax before the tumor board meeting so we could compare the MedMax-generated outcomes with those of the tumor board. The decisions made through MedMax were made in a non-clinical setting and did not influence the actual decision-making process.

### 2.2. Methods

#### 2.2.1. Structure

**Figure 1:**
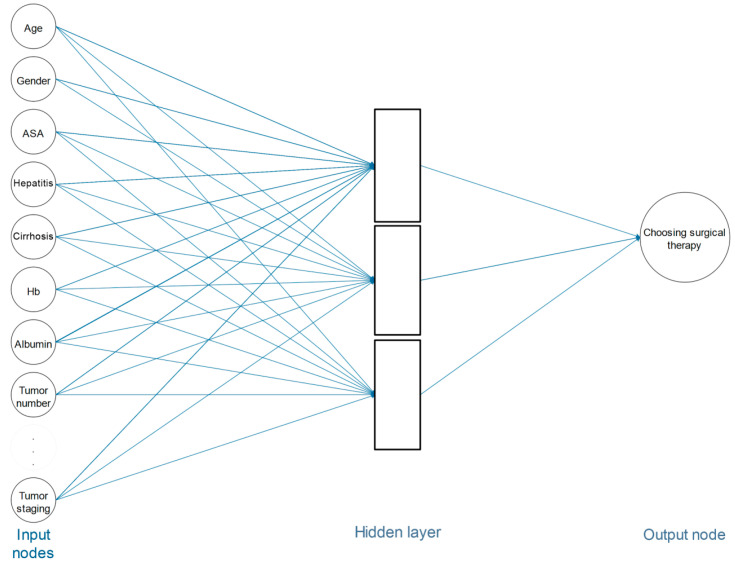
Pattern of decision-making process in treatment of patients with ihCC.

The model was composed of two main parts: (1) factors associated with choosing the best treatment and (2) the phases and steps that are necessary for therapeutic decision making based on these factors. The correlation between the clinical factors and the decision-making process was established with due attention to the characteristics previously defined for each parameter. As shown in Figure 1, the network’s structure consists of nodes (representing clinical factors) and edges (representing connections between these factors and the decision-making process). For instance, the nodes symbolize the various relevant factors in the decision-making process, while the edges represent dependencies and interactions between these factors and the decision pathway. This structure allows the transformation of input data into predictive values using internal representations. Additionally, the network comprises three primary components:

- Input Nodes: Forming the first layer of the network, responsible for collecting initial data inputs.
- Hidden Layers: Positioned between the input and output layers, processing input data through weighted connections and activation functions to model complex relationships.
- Output Nodes: Representing the final layer of the network, providing predictions based on the processed inputs from the hidden layers.

#### 2.2.2. Definitive Factors

The heterogeneous factors obtained from the literature review and expert opinions (Supplementary File S1) were classified into the following eight categories: baseline data, medical history, diagnostic clarifications, diagnostic evaluation, surgical planning, tumor resectability, postoperative care, and oncological results. The decision-making processes in this framework are divided into the following phases:

- Diagnostic Phase (Phase I)

This initial phase involves a comprehensive evaluation of laboratory parameters and tumor markers. Additionally, imaging, endoscopic procedures, and histopathological analyses are performed to confirm the diagnosis and provide a solid foundation for subsequent steps.

- Staging Phase (Phase II)

The focus of this phase is the detailed application of imaging and endosonographic techniques. These are used to accurately determine the disease stage according to the internationally recognized TNM staging system.

- Treatment Planning Phase (Phase III)

During this phase, various treatment options are considered, including surgical procedures, chemotherapy, radiotherapy, and combination therapies. These options are discussed and evaluated based on the staging results, surgical resectability, assessment of liver function, and other patient-specific factors.

- Therapy Phase (Phase IV)

This final phase centers on the practical implementation of the chosen treatment methods, continuous monitoring of therapeutic success, and evaluation of treatment response.

**Figure 2:**
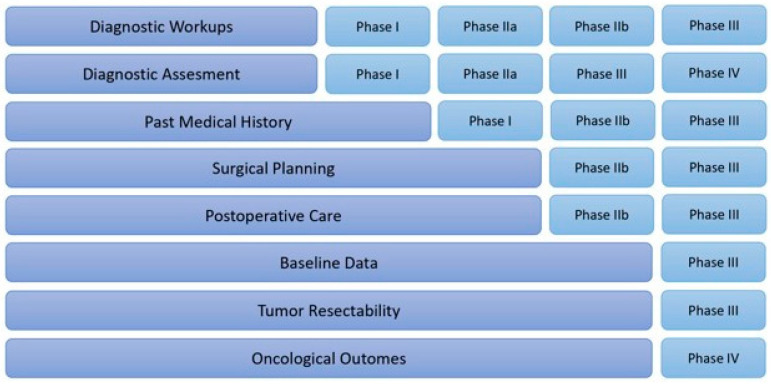
Classification of the parameters used for matrix design and relevance to different steps.

Based on this structure, we developed the phases of our matrix model, which are clinically significant for selecting the optimal therapeutic approach. Particular emphasis was placed on the different surgical strategies. Our model involves careful consideration of factors crucial for individualized treatment strategies, including tumor characteristics, patient profiles, and the potential benefits of surgical interventions, which has been summarized in Figure 2. This figure demonstrates which characteristics and information were used at each phase of the matrix.

To automate and subsequently evaluate the review of parameters for each patient, the type of each parameter was defined based on nominal, continuous, ordinal, and discrete subtypes. The value and range of each factor was also described to eliminate any deviants (outliers). Most of the data included in the model were collected using a dichotomous zero and one method. However, for continuous parameters, a cut-off value was specified to facilitate the decision-making process. For multinominal parameters, a specific numerical value was specified for each grade or level, which could be used either by specifying the value of each level and grade of classification or by classifying by a predefined cut-off value. Furthermore, the parameters were only used in some stages, so the parameters related to each stage were separated from the others according to relevance. This means that, when checking each phase, the matrix evaluated only those parameters that were defined as relevant. It is important to note that the parameters could be used multiple times across different phases. Each parameter was used in at least one phase, and its value could vary each time it was used, depending on the specific goals of that phase. Consequently, the limits set for a parameter could change if it was employed in more than one phase. Three sources were used to establish these limits for each parameter.

Once the phases and their relevant factors, including specific threshold values, were defined, the individual factors were categorized according to their data type and threshold values relevant to each phase. Using the traffic light model, each category was assigned a color (green, yellow, red) to clarify, visualize, and—most importantly—make the clinical relevance and risk status machine-readable.

The machine can now initiate data analysis by reviewing all relevant factors in the first phase. If each factor is assigned a green or yellow light, the patient can proceed to the next phase of evaluation. If a red light is assigned, the evaluation is stopped. At the conclusion of a successfully completed evaluation, all yellow flags are listed and made available for further risk assessment. During the development of our decision-making matrix, it became evident that some factors that currently lead to inoperability—and, thus, exclude patients from the matrix—might be resolved through additional evaluations and treatments. For this reason, it was decided to introduce a new indicator color: orange. This color allows affected patients to be temporarily removed from the matrix. Once the criteria for operability are met, these patients can be reintroduced into the matrix and re-evaluated. However, since tumors are dynamic and progressive in nature, the patient must restart the matrix analysis from the beginning, depending on the duration of further evaluations.

**Figure 3:**
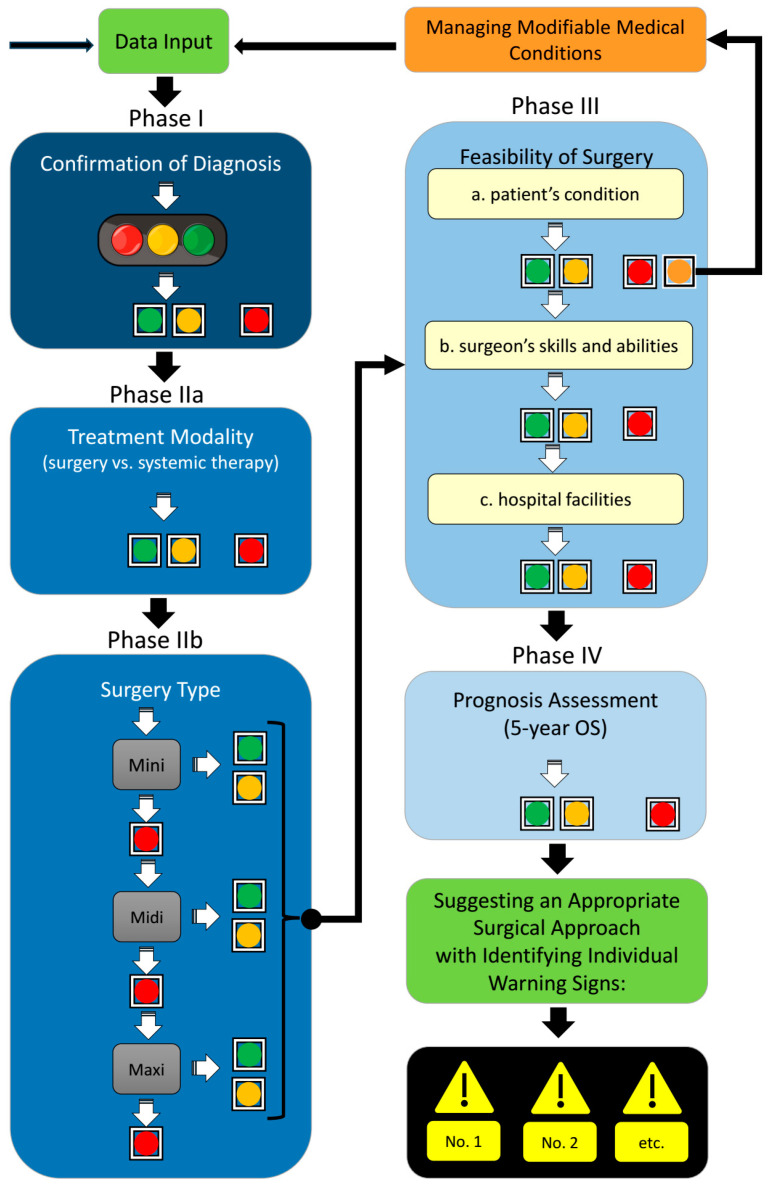
Schematic representation of the matrix operation and description of the traffic light model. (Abbreviations: OS: overall survival).

Figure 3 illustrates the functionality of the traffic light model following its integration into the decision-making matrix. This visualization facilitates understanding of the dynamic processes within the matrix and the potential decision pathways based on factor evaluations.

#### 2.2.3. Decision-Making Phases

In our institute, tumor boards make therapeutic decisions based on confirmation of the diagnosis, the therapeutic modality, and the feasibility of the modality. We categorized this approach into four phases: (1) verifying the ihCC diagnosis, (2) choosing between systemic therapies and surgery and determining the type of surgery, (3) assessing the surgical approach according to the patient’s condition, surgeon’s skills and abilities, and hospital facility, and (4) determining the prognosis of surgery (5-year overall survival) (Figure 3).

Although each phase of the proposed model is a completely independent stage in the patients’ evaluation process, the model benefits from a stepwise structure. In other words, the decision would be made separately in each particular phase. However, the outcome of the earlier phase is the main prerequisite for acceptable decision making, and it defines whether each patient qualifies for the next step and whether they would benefit from surgical treatment.

## 3. Results

### 3.1. Primary Results of MedMax

#### 3.1.1. Factor Extraction

**Figure 4:**
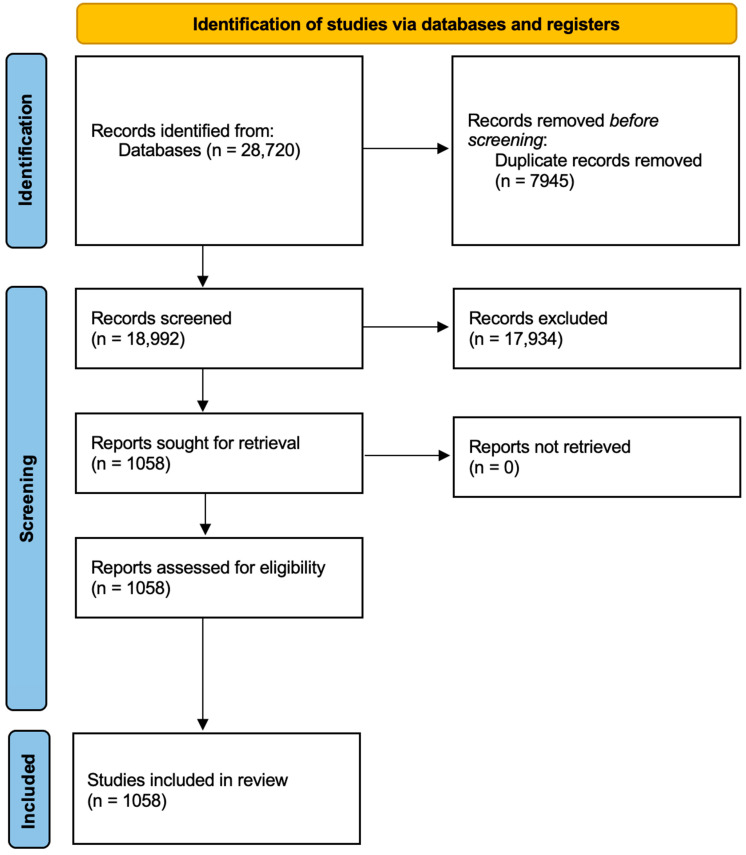
Prisma flowchart of the studies evaluated for extraction of parameters.

Electronic searches of the Medline and Web of Science databases retrieved 28,387 studies. After removing 7945 duplicates, 18,659 records were excluded by a primary screening of titles and abstracts, and 725 manuscripts were excluded by a detailed assessment of full texts for eligibility. Finally, 1058 studies were included in the quality assessment (Figure 4).

After analysis of the results obtained from our literature search, 45 parameters were found to be relevant to decision making in the treatment of ihCC. Demographic and clinicopathological data were age; sex; body mass index; American Society of Anesthesiologists score; preoperative levels of CA19-9, carcinoembryonic antigen, alpha-fetoprotein, c-reactive protein, total and direct bilirubin, albumin, glutamate–pyruvate transaminase, glutamate oxaloacetate transaminase, gamma-glutamyltransferase, and alkaline phosphatase; prothrombin time; preoperative complete blood count; albumin-bilirubin (ALBI) grade calculated by the following formula: [log10 bilirubin (µmol/L) × 0.66] + [albumin (g/L) × −0.085]; cirrhosis (categorized by the Child–Pugh score); heart failure (categorized by the New York Heart Association classification); chronic obstructive pulmonary disease (categorized by the Global Initiative for Chronic Obstructive Lung Disease classification); chronic hepatitis B or C; end-stage renal failure; preoperative glomerular filtration rate; cholangitis; preoperative histopathologic sampling; type of resection (minor, intermediate, or major); location of the tumor; preoperative lymph node status; number and size of the tumor; metastasis and possible primary site; portal hypertension; severity of ascites; severity of fatty liver; peritoneal carcinomatosis; predicted residual liver volume; major vascular structure (defined as invasion of first and second order branches of portal vein or hepatic arteries or as invasion of one or more of the three hepatic veins); and the availability of postoperative intensive care.

Regarding the ALBI grade, patients were divided into three groups as follows: grade 1 ≤ −2.60, −1.39 ≥; grade 2 ≥ −2.60; and grade 3 ≥ −1.39. Major hepatectomy was divided into three subgroups: left extended hepatectomy, right extended hepatectomy, or mesohepatectomy. Extended hepatectomy was defined as the resection of five or more Couinaud segments. Mesohepatectomy was defined as the resection of central liver segments, including Couinaud segments IVa, IVb, V, and VIII. Intermediate hepatectomy was divided into four subgroups: left and right hemihepatectomy (i.e., resection of the left or right half of the liver) and left and right mesohepatectomy (i.e., resection of Couinaud segments V and VIII or IVa and IV). Minor hepatectomy was divided into two subgroups: segmentectomy and bisegmentectomy.

#### 3.1.2. Expert Opinion

Twenty-five experienced surgeons were included in this survey. They rated the listed factors using a Likert scale. All 45 factors, plus 18 different permutations related to tumor location that affect concordant curable liver resection, received 5 points and were used to create the matrix.

#### 3.1.3. Phases of Decision Making

**Figure 5:**
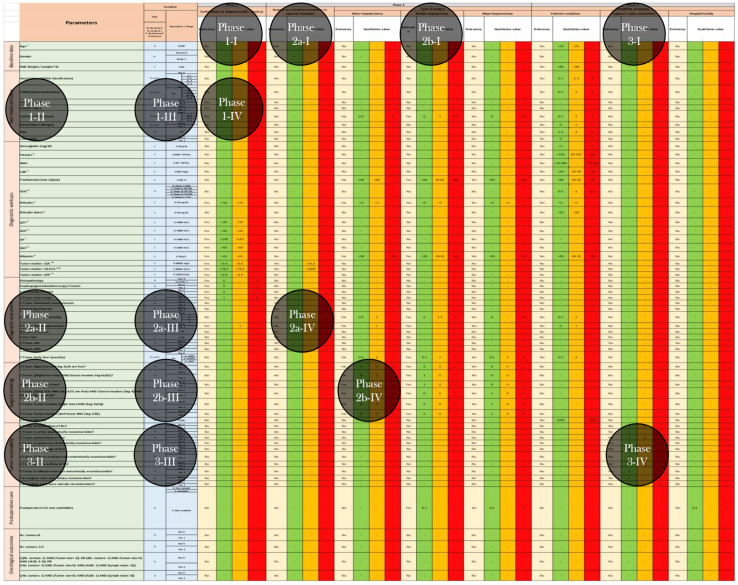
Design of the decision-making matrix including parameters and phases.

**Figure 6:**
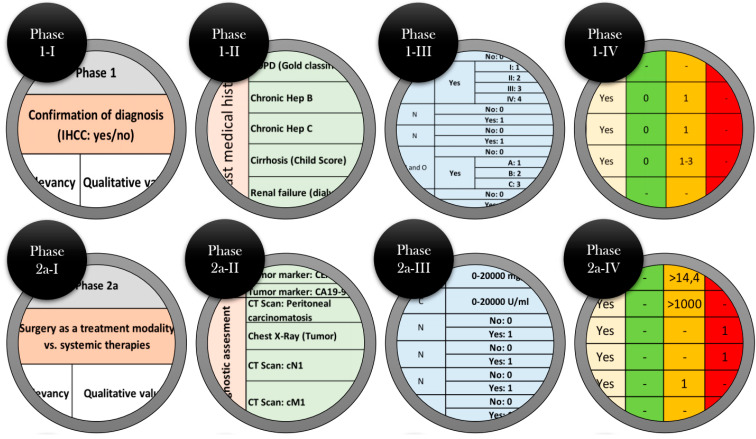

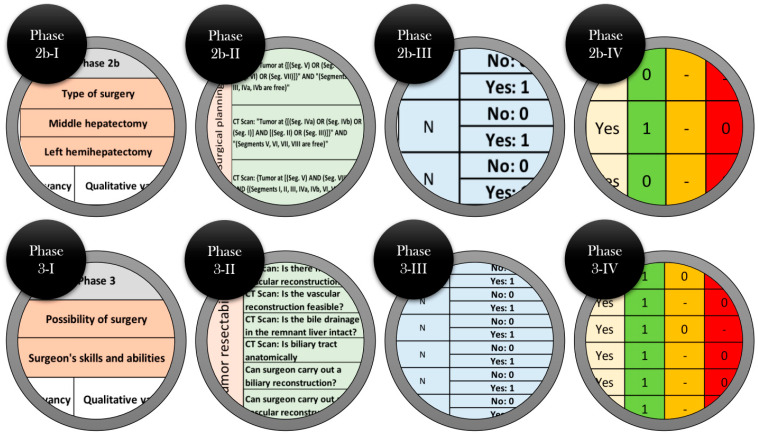
Overview of the phases and subphases of the matrix.

Considering the parameters involved in each of the three decision-making phases, a multiphase evidence-based decision matrix for the surgical treatment of ihCC was constructed. The following overview, presented in Figure 5 (high quality version in Supplementary File S1) illustrates the MedMax matrix in its entirety, including the integrated phases and their associated factors. This matrix provides a comprehensive overview of the structure and systematic relationships within the model. To ensure a more detailed and visually clear representation, the highlighted circles within the matrix are shown separately and magnified in Figure 6. These detailed views are intended to provide a deeper understanding of the multi-layered and multi-phased nature of the MedMax matrix, as well as its dimensions.

The decision phases were as follows:

(1) Confirm Suspected ihCC According to the Paraclinical and Clinical Workups (Laboratory, Imaging, and Histopathological Findings)

**Figure 7:**
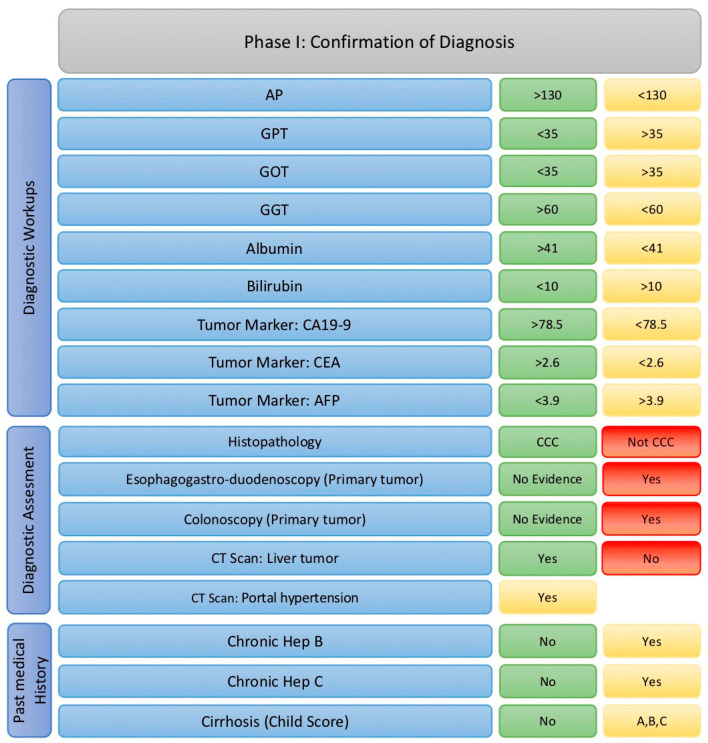
Factors implemented in phase I with their cut-offs adapted to traffic light model. (Abbreviations: AFP: alpha-fetoprotein; AP: alkaline phosphatase; CEA: carcinoembryonic antigen; GGT: gamma-glutamyltransferase; GOT: glutamate oxaloacetate transaminase; GPT: glutamate–pyruvate transaminase).

In this phase, either the diagnosis of ihCC is confirmed, or a high suspicion of the condition is established. The relevant patient characteristics at this stage include:

- Medical History,
- Diagnostic Workups (laboratory investigations), and
- Diagnostic Assessments (as illustrated in Figure 7).

Medical History

Three prior conditions are particularly relevant: the presence of hepatitis B, hepatitis C, and liver cirrhosis. A negative history of these liver conditions strengthens the suspicion of ihCC and is, therefore, classified as green. While the presence of these conditions does not rule out ihCC, they are marked yellow since they are associated with other liver pathologies and may complicate the diagnosis.

Relevant Laboratory Parameters

The key laboratory values assessed during this phase include alkaline phosphatase (AP), glutamate oxaloacetate transaminase (GOT), glutamate–pyruvate transaminase (GPT), gamma-glutamyl transferase (GGT), albumin, total bilirubin, and tumor markers CA 19-9, CEA, and AFP (alpha-fetoprotein). High values of AP, GGT, albumin, CA 19-9, and CEA are favorable for an ihCC diagnosis and are classified as green. Conversely, low values of GOT, GPT, bilirubin, and AFP are advantageous for the diagnosis and are also marked green. Intermediate or borderline values that do not rule out a suspicion of ihCC are marked yellow.

Relevant Diagnostic Procedures

The diagnostic procedures considered include histopathological, imaging, and endoscopic evaluations, such as computed tomography (CT), esophagogastroduodenoscopy (EGD), and colonoscopy.

(A) Histopathological Findings: Results indicating intrahepatic cholangiocarcinoma (ihCC) are marked green. Results pointing to other liver tumors are marked red. Unclear findings or suspicion of mixed tumor types are marked yellow.
(B) Endoscopic Investigations: Identification of a primary tumor via endoscopy is marked red.
(C) Imaging (CT): CT findings showing malignancy criteria consistent with ihCC are marked green. CT findings indicating other liver tumors are marked red.

**Figure 8:**
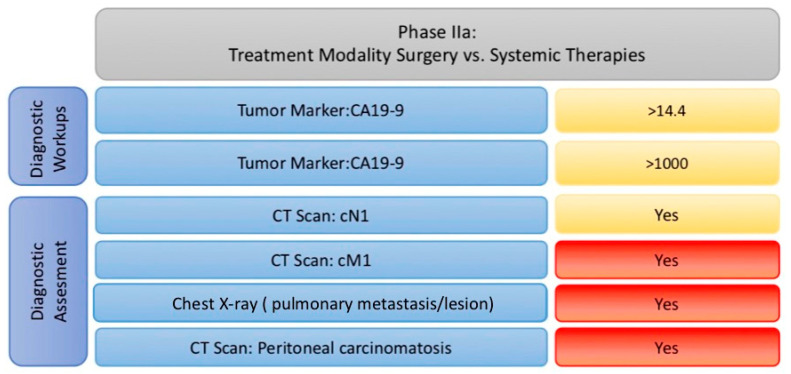
Factors implemented in phase IIa with their cut-offs adapted to traffic light model. (Abbreviations: CA 19-9: carbohydrate antigen; CT: computer tomography).

This phase focuses on systematically classifying relevant data points using a traffic light system to streamline the diagnostic process and ensure a structured evaluation leading to an accurate diagnosis or a well-supported suspicion of ihCC. If the patient exits the matrix at this stage with a red light, they should ideally be transferred to another matrix and evaluated there according to the new diagnosis. If the patient achieves a yellow or green light for all parameters, they proceed to the second phase of the matrix.

(2a) Determine the most appropriate treatment modality (surgery or systemic therapy) after evaluating distant metastases (Figure 8).

Phase 2 consists of two steps aimed at selecting the appropriate treatment modality, considering paraclinical examinations, the extent of locoregional disease, and the evaluation of distant metastases. The tumor staging was carried out according to the American Joint Committee on Cancer (AJCC) TNM system [16]. If resection emerges as the preferred therapy, this phase also determines the type of surgical intervention and assesses the surgical risk based on the planned extent of resection.

In the first step of Phase II, two criteria are used to assess the extent of locoregional disease and metastasis spread: tumor markers (CEA, CA 19-9) and imaging findings (CT of the abdomen/thorax and, if necessary, chest X-ray). This ultimately serves to evaluate resectability.

- Red Light: If distant or peritoneal metastases are detected in contrast-enhanced CT or chest X-ray, the patient is marked red.
- Yellow Light: Extensive tumor spread, which could endanger resectability and is associated with increased perioperative morbidity and mortality, is flagged with very high tumor marker levels (CEA > 14.4 ng/L and CA 19-9 > 1000 U/mL) or the involvement of regional lymph nodes.

**Figure 9:**
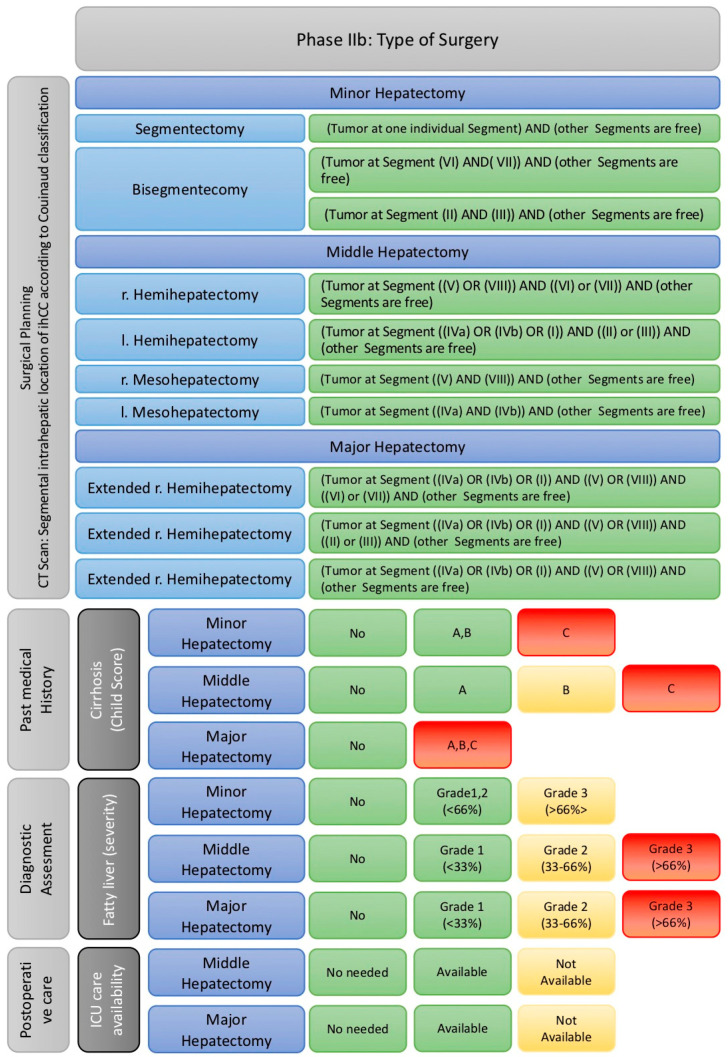
Classification and categorizing the types of liver resection and surgical planning in Phase IIb as well as restricting factors based on the type of resection adapted to traffic light model.

A clear overview of these classifications is provided in Figure 8. If the patient is deemed eligible for surgery as a primary treatment, they proceed to the second step of Phase 2.

(2b) Determine the type and extent of surgery if the patient is a candidate for surgical treatment (Figure 9).

The second step of Phase II is divided into two interconnected layers, each building upon the other. The first layer must be thoroughly evaluated and completed to establish a solid foundation for analyzing the second layer. However, each layer is independently designed with its own matrix to address specific challenges and goals. The matrix includes the following core components, detailed in Figure 9:

i. Determining the appropriate anatomical liver resection.

Based on tumor localization in imaging studies and considering the Brisbane and Couinaud classifications, the variations in liver involvement have been precisely identified. These variations have been matched to their corresponding anatomically appropriate surgical procedures and are grouped into three main categories: Major, Intermediate, and Minor Hepatectomy. These main categories are further divided into 18 specific subcategories representing various surgical approaches.

To convert this complex information into a machine-readable format, the different types of liver resections were systematically recorded as strings and then encoded into arrays as follows:

- Minor Hepatectomy
  - Segmentectomy:
    “{Tumor present only in one segment} and {other segments are free}.”
  - Bisegmentectomy:
    “{Tumor present only in segments VI and VII} and {all other segments are free}.” “{Tumor present only in segments II and III} and {all other segments are free}.”
- Intermediate hepatectomy
  - Right Hemihepatectomy:
    “{Tumor present either in segment V or VIII} and {either in segment VI or VII} and {all other segments are free}.”
  - Left Hemihepatectomy:
    “{Tumor present either in segment IVa or IVb or I} and {either in segment II or III} and {all other segments are free}.”
  - Right Mesohepatectomy:
    “{Tumor present only in segments V and VIII} and {all other segments are free}.”
  - Left Mesohepatectomy:
    “{Tumor present only in segments IVa and IVb} and {all other segments are free}.”
- III. Major Hepatectomy
  - Extended Right Hepatectomy:
    “{Tumor present in segment VI or VII} and {in segment V or VIII} and {in segment IVa or IVb or I} and {all other segments are free}.”
  - Extended Left Hepatectomy:
    “{Tumor present in segment IVa or IVb or I} and {in segment II or III} and {in segment V or VIII} and {all other segments are free}.”
  - Mesohepatectomy:
    “{Tumor present in segment IVa or IVb or I} and {in segment V or VIII} and {all other segments are free}.”

During data analysis by the machine, the traffic light model is applied in a modified way in this context. MedMax must carefully examine all fields within the matrix to identify a suitable permutation that offers the potential for a tumor-free resection margin (R0 resection). If a red light is triggered for a field, the patient is not immediately excluded. Instead, MedMax continues to analyze other fields to find a suitable intervention leading to a green light, thereby enabling a potentially successful treatment. If the matrix cannot identify an appropriate liver resection, the evaluation is halted. In this case, the patient is marked red and excluded from further surgical attempts.

If the matrix identifies a suitable permutation, the process continues into the second layer of Phase IIb, where the benefits of the planned liver resection are assessed considering all risk factors.

ii. Assessing the risk associated with the chosen resection.

In the second layer of Phase IIb, a comprehensive risk assessment is conducted to evaluate the feasibility of the planned liver resection. This evaluation considers three central factors: degree of liver cirrhosis, severity of liver steatosis, and availability of adequate postoperative intensive care.

- Liver Cirrhosis:
  ○ Child C: Immediate stop for any resection (red light).
  ○ Child B:
    ■ Minor Hepatectomy: No risk, marked green.
    ■ Intermediate Hepatectomy: Restriction present, marked yellow.
- Liver Steatosis:
  ○ Grade 3 (≥66%):
    ■ Major and Intermediate Hepatectomies: Contraindicated, marked red.
    ■ Minor Hepatectomy: Warning, marked yellow.
  ○ Grade 2 (33–66%):
    ■ Major and Intermediate Hepatectomies: Warning, marked yellow.
    ■ Minor Hepatectomy: Marked green.
- Postoperative Intensive Care:
  ○ Insufficient care:
    ■ Minor Hepatectomy: No issue, marked green.
    ■ Intermediate Hepatectomy: Warning, marked yellow.
    ■ Major Hepatectomy: Exclusion, marked red.

If the matrix fulfills all criteria for an appropriate liver resection without triggering a red light for the above limiting factors, the patient advances to the third phase. Otherwise, surgical therapy is deemed infeasible, and the patient is directed to the matrix for palliative or potentially neoadjuvant therapy options.

(3) Evaluate the feasibility of surgery depending on the general condition of the patient, the skills of the surgeon, and the facilities of the hospital

**Figure 10:**
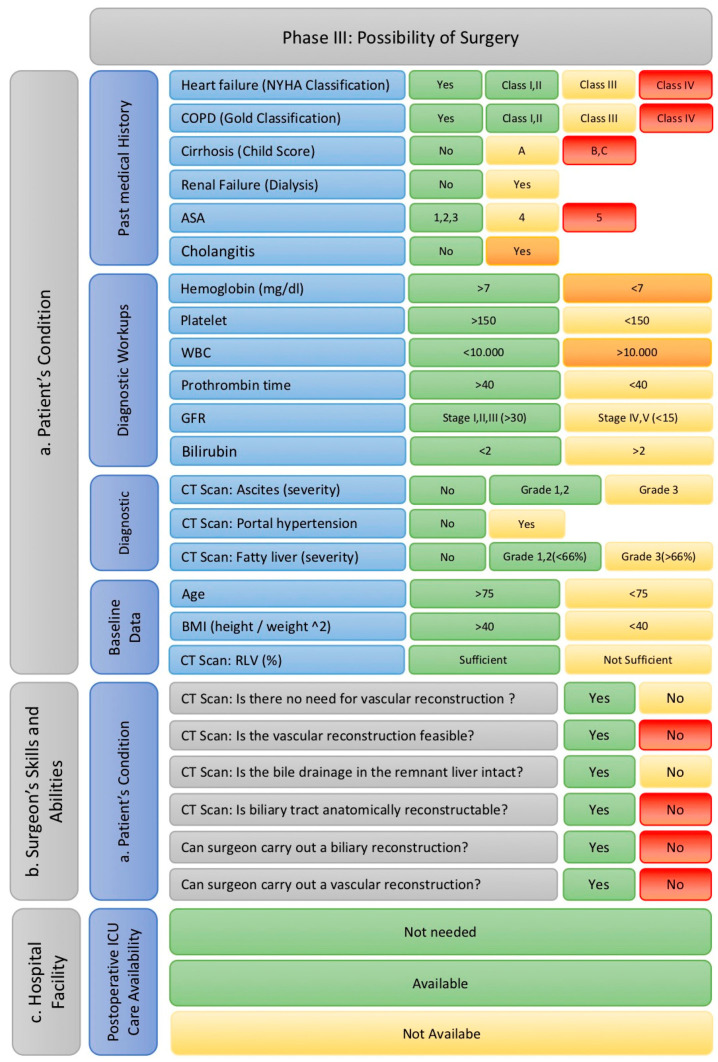
Factors implemented in phase III with their cut-offs adapted to traffic light model. (Abbreviations: ASA: American Society of Anesthesiologists; BMI: body mass index; COPD: chronic obstructive pulmonary disorder; GFR: glomerular filtration rate; WBC: white blood cells).

In this phase, the feasibility of the proposed surgical intervention is assessed based on three critical aspects: the patient’s health condition, the qualifications and skills of the surgeon, and the hospital’s facilities, as illustrated in Figure 10.

To evaluate the patient’s ability to undergo the surgery determined in the previous phase, the matrix considers several factors. The patient’s medical history plays a significant role, including heart failure (classified using the NYHA system), COPD (evaluated with the Gold classification), liver cirrhosis (according to the Child classification), chronic kidney failure, the ASA classification, and the presence of acute cholangitis. Patients with severe conditions, such as NYHA stage IV heart failure, Gold stage IV COPD, Child B or C liver cirrhosis, or an ASA classification of V, are excluded and flagged with a red light. Moderate conditions, like NYHA stage III heart failure, Gold stage III COPD, Child A liver cirrhosis, or ASA classification IV, are marked as warning signs with a yellow light. Acute cholangitis, however, requires immediate attention and leads to a temporary exclusion with an orange light until further assessments and treatments are conducted.

Laboratory parameters are also examined, including hemoglobin, platelets, leukocytes, total bilirubin, PTT, and GFR. Mild abnormalities, such as thrombocytopenia, hyperbilirubinemia, prolonged PTT, or reduced GFR (indicative of chronic kidney disease stages 4 or 5), are considered warning signs and flagged with yellow. Severe deviations, like anemia (Hb < 7 g/dL) or leukocytosis (>10,000/μL), result in an orange light, requiring temporary exclusion until corrective measures are taken. Baseline patient data, such as age and BMI, are also evaluated. Advanced age (≥75 years) and high BMI (≥40 kg/m^2^) are flagged as potential risks with a yellow light, indicating the need for careful consideration. Diagnostic assessments, including CT findings of ascites, liver steatosis, portal hypertension, and insufficient residual liver volume (RLV), are similarly marked with a yellow light if they suggest significant risks.

The surgeon’s qualifications and skills are assessed in the context of the complexity of the planned surgery. Parameters like vascular involvement (e.g., artery, portal vein, or inferior vena cava) and challenges in post-resection biliary drainage are closely examined. If these reconstructions are not feasible, the patient is excluded with a red light. Even if reconstruction is technically possible but the surgeon lacks the necessary expertise, the patient is excluded. However, if the surgeon possesses the required qualifications but the case remains high-risk due to factors like vascular involvement or biliary complications, the patient is flagged with a yellow light to indicate heightened risk.

The hospital’s facilities, particularly the availability of perioperative intensive care, are also critical. A lack of adequate intensive care support is flagged with a yellow light, highlighting the elevated risk but not necessarily leading to the cancelation of the operation.

If the patient receives a red or orange light for any of these aspects, surgery is not possible. A red light results in the patient being directed to the palliative therapy matrix to explore alternative treatment options, while an orange light indicates the need for additional evaluations or therapies. Once the identified issues are resolved, the patient can be re-entered into the surgical matrix for reassessment. On the other hand, if the patient receives only green or yellow lights, they successfully pass through the matrix and receive a final evaluation. If surgery is confirmed as the appropriate treatment modality, the most suitable curative surgical approach is determined. Cumulative warning signs are also highlighted to assist in further risk stratification and final decision making.

**Figure 11:**
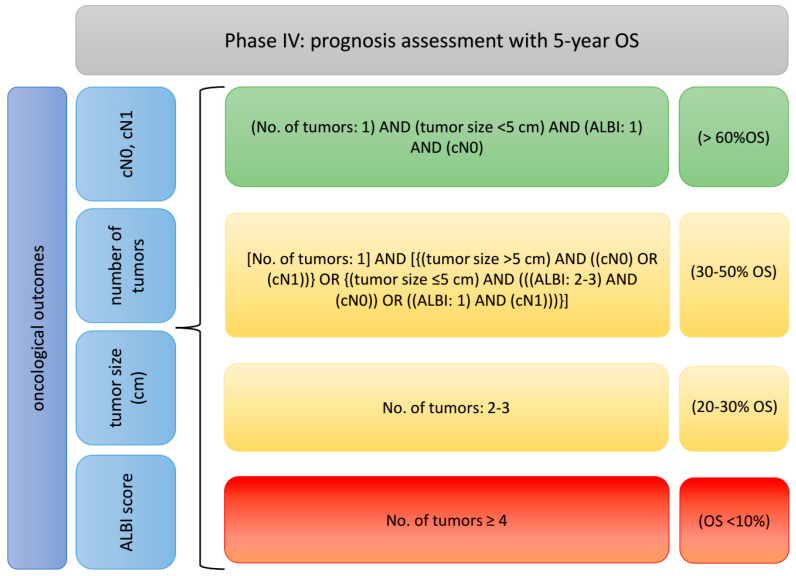
Factors implemented in phase IV with their cut-offs adapted to traffic light model. (Abbreviations: OS: overall survival).

(4) Predict the prognosis using 5-year overall survival (Figure 11).

### 3.2. Analysis of the Decisions Made by MedMax

All patients with suspected ihCC who were candidates for discussion at the interdisciplinary tumor board were processed by the MedMax decision-making model. During the 12-month study period, 44 (15.3%) out of 288 patients were included and all necessary data were obtained.

In phase I, the matrix was able to properly confirm or rule out ihCC in all patients. In phase II, not only the appropriate treatment modality but also the type of surgical approach was determined in all patients. In the first step of phase II, MedMax selected those patients who would benefit from surgical treatment with an accuracy of 100%. In the second step of phase II, the matrix recommended patients for surgery with an accuracy of 100% and was able to determine the type of surgery with an accuracy of 77.3%. In phase III, the matrix evaluated the feasibility of surgery concerning the clinical condition of the patient, the skills of the surgeon, and the facilities of the hospital, and these results were completely compatible (100%) with those of the internal preoperative assessment in our hospital. The outcomes of the decision-making process at each phase are presented separately for each patient in Figure 9. Decisions generated by MedMax were consistent with those made at tumor board sessions and showed an accuracy of 100%. The compatibility between the decisions generated by MedMax and those of the tumor board confirms the high accuracy of the proposed model in making treatment decisions for patients with ihCC.

## 4. Discussion

HPB surgery is still considered a challenging field of surgery, based on its association with meticulous and precise works around the complex vascular and biliary structures, demanding accurate dissections. Also, primary liver tumors are not easily discovered at their initial stage; therefore, lack of early diagnosis leads to more limited therapeutic options [17,18,19]. Accordingly, ihCC is the second most common primary malignant liver tumor, which is located within hepatic parenchyma and accounts for 10% of primary hepatic malignancies cases [20,21]. According to the most recent guidelines, including the National Comprehensive Cancer Network (NCCN) Clinical Practice Guidelines in Oncology (Version 4.2024—29 August 2024), the treatment strategy for ihCC should primarily be determined by tumor resectability. These guidelines highlight surgery as the only curative treatment option, while other modalities, such as arterial and locoregional therapies, are reserved for patients who are not candidates for curative surgery or require downstaging for other therapies. Therefore, liver resection can be considered as the primary curative treatment for patients with ihCC, and timely decisions on the best treatment approach is the only way to extend survival in these patients [22,23]. These decisions must consider the individual patient, as well as the features and manifestations of their disease. For this, several factors should be considered in the decision-making process, but these factors are difficult to determine because “big data” cannot be processed and interpreted by traditional techniques [24,25,26].

In this study, we proposed a four-phase MedMax model that (1) confirms the ihCC tumor diagnosis, (2) selects those patients who will benefit from surgical treatment, (3) evaluates the possibility of liver resection based on patients’ clinical condition, and (4) predicts the 5-year overall survival of the patients after curative liver resection. The second phase was also able to determine the best liver resection option based on the tumor characteristics and liver function. The MedMax model signifies a major advancement in clinical decision making for ihCC by integrating a wide range of clinically relevant factors. It encompasses all phases from diagnosis to indication setting and surgical planning, including risk stratification. This comprehensive and unified approach is unique in the existing literature, addressing gaps commonly found in other models. Previous approaches often focus on isolated aspects, such as diagnostics, imaging, or surgical and chemotherapeutic interventions, without providing a connecting perspective. MedMax overcomes this fragmentation by working holistically and interdisciplinarily, ensuring seamless integration across all clinical decision phases. Despite the low number of patients, the initial validation revealed promising efficacy for the MedMax model in defining the best and individualized treatment for patients. Furthermore, we used MedMax to generate treatment decisions before the tumor board made their decisions, so were able to compare MedMax decisions with those of the tumor board prospectively.

To date, most prognostic models developed to support therapeutic decision making or to predict outcomes in patients undergoing liver resection have been specifically designed for those undergoing curative resection. These models predominantly rely on histopathological factors that are only available postoperatively. For instance, the nomograms developed by Hyder et al. [27] and Wang et al. [28] incorporate microvascular invasion, while models such as the MEGNA prognostic score [29] and the early ihCC prognostic model include factors like histological lymph node status and tumor differentiation, respectively. While these models demonstrated promising initial results, the nomograms by Wang et al. and Hyder et al. failed to achieve validation in external cohorts [30,31,32]. The Fudan score is the only model that does not require histopathological data, making it applicable in a broader clinical context [33]. However, despite its high discriminative ability, external validation studies have shown only moderate accuracy in predicting survival outcomes [34]. These limitations highlight the ongoing need for robust and externally validated prognostic models that can reliably guide clinical decision making. Our MedMax model identified many parameters that were crucial for making decisions about the treatment of patients with ihCC according to the scientific literature and expert opinions. Therefore, MedMax seems to be able to choose the best treatment based on patient benefit. However, it should be noted that this is only an early version of a decision-making platform and that it could be expanded to other liver tumors and other indications of liver surgery in the future.

Previous studies have shown that this decision matrix methodology is both straightforward to interpret and highly accurate [35,36,37]. When used for individual decision making, the matrix adapts to responses to specific questions, guiding different variables toward the final decision. Rather than following a linear approach, the algorithm used in this model identifies complex relationships among variables to provide personalized treatment recommendations. The model can also identify cumulative high-risk parameters (flagging them with a yellow traffic light), which can aid discussions on the risks and benefits of surgery while pinpointing those patients who would benefit from preoperative strategies to minimize perioperative risk. Although some preoperative factors are immutable at the patient level, various steps can be taken to reduce the likelihood of certain complications. Unlike data processing using a Blackbox approach [38], decision making through MedMax offers a clear, transparent path for personalized treatment, effectively handling complex data and tailoring the decision-making process to each individual [39,40].

Another advantage of MedMax is that it is dynamic so can be extended into more accurate and detailed versions by incorporating new evidence and refining different phases. It can be expanded and updated without significant structural changes. Unlike other decision-making models like the Classification and Regression Tree (CART) algorithm, MedMax has a flexible structure so can feasibly be expanded. The simple structures of algorithms like CART often restrict changes because alterations significantly disrupt their framework [41,42]. The different phases of MedMax act as independent blocks, enabling the matrix to be easily modified and updated without affecting the other phases. This ensures that the system can evolve as medical knowledge and practices advance. Our model contains many parameters that play roles in decision making for ihCC patients according to the literature and experts in the field. Therefore, in contrary to data processing via Blackbox, the decision-making process via MedMax provides a strong and transparent pathway for individualized treatment. However, no accurate and definitive cut-off values have been reported in the literature for all parameters in our matrix structure. The MedMax structure has been transformed into a machine-readable format, enabling the integration of the traffic light model. This integration facilitates comprehensive risk stratification, cumulatively considering all phases of the decision-making process. This not only achieves a differentiated risk assessment but also paves the way for the future integration of artificial intelligence (AI). AI could further optimize personalized decision making, improve the precision of risk factor evaluations, and enable well-founded therapeutic decisions. Such advancements significantly contribute to the enhancement of personalized medicine and support data-driven clinical decision processes.

When interpreting the results of this study, certain limitations must be considered. The retrospective design of the study limits the generalizability of the MedMax model to specific contexts. Additionally, MedMax is currently based on data collected from a single center, which restricts its external validity. Although an internal validation with retrospective data of patients undergone liver resection for ihCC is planned, future multicenter studies that implement the ihCC-specific MedMax model could facilitate a more detailed comparison of therapeutic approaches across different institutions and help identify better-adapted treatment options with enhanced oncological surgical reliability for each center. Furthermore, the current algorithm is restricted to preoperative variables. Given the critical role of intraoperative factors in final decision making, these should be integrated into future iterations of MedMax to enhance the model’s predictive accuracy and applicability. Atypical liver resections were also excluded from the development of surgical options due to their diversity and lack of specificity. This led to the misclassification of some potentially operable multifocal bilobar liver involvements as inoperable, slightly affecting the predictive performance of the matrix. Another limiting factor is the relatively small sample size, and further studies are required to externally validate the model. Through multicenter collaborations, a larger number of patients could be included in clinical studies, supporting broader validation and adaptation of such algorithms to diverse clinical environments.

Finally, further developments are needed to enhance the clinical and practical application of MedMax as a supplementary tool for tumor boards. We believe that MedMax should provide the basis for developing an expert system with a user-friendly interface for individualized decision making in hospitals and clinics.

## 5. Conclusions

The presented multiphase decision matrix introduces an innovative model that, for the first time, provides a seamlessly integrated diagnostic and therapeutic pathway for patients with ihCC. This matrix offers a structured risk assessment at each stage of the decision-making process, facilitating the integration of patient-specific, surgical, oncological, and logistical factors to refine treatment choices. By presenting oncologically optimal and surgically feasible options, the model supports a nuanced and personalized approach in oncologic liver surgery. The innovative combination of robust clinical data with patient-specific risk stratifications provides a solid foundation for optimizing preoperative planning processes, significantly improving the efficiency and outcomes of liver surgery.

## Data Availability

Data of the present study can be made available upon logical request to the corresponding author and after evaluation of the request by the ethics committee of the university.

## Supplementary Materials

The following supporting information can be downloaded at: https://www.mdpi.com/article/10.3390/cancers17010052/s1, Supplementary Table S1. Factors associated with the therapeutic decision-making in patients with intrahepatic cholangiocarcinoma and outcomes of evaluation by experts. Supplementary Figure S1. Design of the decision-making matrix including parameters and phases. References [43,44,45,46,47,48,49,50,51,52,53,54,55,56,57,58,59,60,61,62,63,64,65,66,67,68,69,70,71,72,73,74,75,76,77,78,79,80,81,82,83,84,85,86,87,88,89,90] are cited in the Supplementary Materials.
