# Supplementary material for: Introducing and Validating the Multiphasic Evidential Decision-Making Matrix (MedMax) for Clinical Management in Patients with Intrahepatic Cholangiocarcinoma
Source: Cancers (Basel). 2024 Dec 27;17(1):52. doi: 10.3390/cancers17010052 (PMC11718823; doi:10.3390/cancers17010052)
Supplement: Supplementary file 1 [file cancers-17-00052-s001.zip › cancers-3268993-supplementary.pdf]

Supplementary file 1

Introducing and Validating the Multiphasic Evidential Decision-Making Matrix (MedMax) for Clinical Management in Patients with Intrahepatic Cholangiocarcinoma

Ali Ramouz [1,2,3], Ali Adeliansedehi [1,2], Elias Khajeh [1,2,3], Keno März [2,4], Dominik Michael [4], Martin Wagner [1,2,5], Beat Peter Müller-Stich [1,2,6], Arianeb Mehrabi [1,2,3] and Ali Majlesara [1,2,3,*]

1 Department of General, Visceral and Transplantation Surgery, University of Heidelberg, 69120 Heidelberg, Germany; ali.ramouz@med.uni-heidelberg.de (A.R.); ali.adeliansedehi@med.uni-heidelberg.de (A.A.); eli-as.khajeh@med.uni-heidelberg.de (E.K.); martin.wagner@med.uni-heidelberg.de (M.W.); beat.mueller-stich@med.uni-heidelberg.de (B.P.M.-S.); arianeb.mehrabi@med.uni-heidelberg.de (A.M.)

2 National Center for Tumor Diseases (NCT) Heidelberg, 69120 Heidelberg, Germany; keno.maerz@dkfz.de

3 Liver Cancer Center Heidelberg (LCCH), University of Heidelberg, 69120 Heidelberg, Germany

4 Division of Computer Assisted Medical Interventions (CAMI), German Cancer Research Center (DKFZ), 69120 Heidelberg, Germany; dominik.michael@dkfz.de

5 Center for the Tactile Internet with Human in the Loop (CeTI), Technical University Dresden, 01069 Dresden, Germany

6 Department of Surgery, Clarunis University Center for Gastrointestinal and Liver Disease, University Hospital and St. Clara Hospital Basel, 4052 Basel, Switzerland

* Correspondence: ali.majlesara@med.uni-heidelberg.de; Tel.: +49-6221-56-8611; Fax: +49-6221-56-4215

Supplementary Table S1. Factors associated with the therapeutic decision-making in patients with intrahepatic cholangiocarcinoma and outcomes of evaluation by experts.

Variables	Study	Average of Likert's scale
**Age**	Vitale et al.(1), Riediger et al.(2)	Most include
**Gender**	Zou et al.(3)	Should include
**BMI**	Merath et al.(4), Yugawa et al.(5), Zhao et al.(6)	Most include
**ASA**	Lopez-Lopez et al.(7), Jehan et al.(8)	Most include
**Hepatitis**	Wang et al.(9)	Most include
**Cirrhosis**	Jesper et al.(10), Capriani et al.(11)	Most include
**Renal failure**	Bagante et al.(12), Yoshikawa et al.(13)	Should include
**Cholangitis**	van Keulen et al.(14)	Most include
**Hemoglobin**	Badawy et al.(15), Giehl-Brown et al.(16)	Most include
**Platelet**	Chatzipanagiotou et al.(17), Chen et al.(18), Margonis et al.(19)	Should include
**CRP**	Ito et al.(20), Nakao et al.(21), Lin et al.(22)	Should include
**Prothrombin time**	Yokoyama et al.(23), Wang et al.(24)	Most include
**GFR**	Bagante et al.(12), Iwasaki et al.(25)	Should include
**Bilirubin**	Munir et al.(26), Kirkland et al.(27)	Most include
**Albumin**	Shen et al.(28), Tsilimigras et al.(29)	Most include
**Transaminases**		Suggested by >50% of surgeons
**Alkaline phosphatase**	Zhang et al.(30)	Should include
**CEA**	Moro et al.(31), Fu et al.(32)	Should include
**CA19-9**	Moro et al.(31), Fu et al.(32)	Most include
**AFP**		Suggested by >50% of surgeons
**Portal hypertension**	Fu et al.(33)	Most include
**Number of tumors**	Jansson et al.(34), Buettner et al.(35), Nassar et al.(36)	Most include
**Tumor size**	Kong et al.(37), Kanu et al.(38)	Most include

**Lymph node metastase**	Jolissaint et al.(39), Zhang et al.(40), Kim et al.(41)	Most include
**Distant metastases**		Suggested by >50% of surgeons
**Tumor location**	Orimo et al.(42), Li et al.(43)	Most include
**Remnant liver volume**	Wang et al.(44), Bednarsch et al.(45)	Most include
**Surgeons' experience**		Suggested by >50% of surgeons
**Vascular infiltration/resection**	Reams et al.(46), Conci et al.(47)	Most include
**Bile duct infiltration/resection**		Should include
**Postoperative intensive care**	Lafaro et al.(48)	Should include

Supplementary Figure S1. Design of the decision-making matrix including parameters and phases.

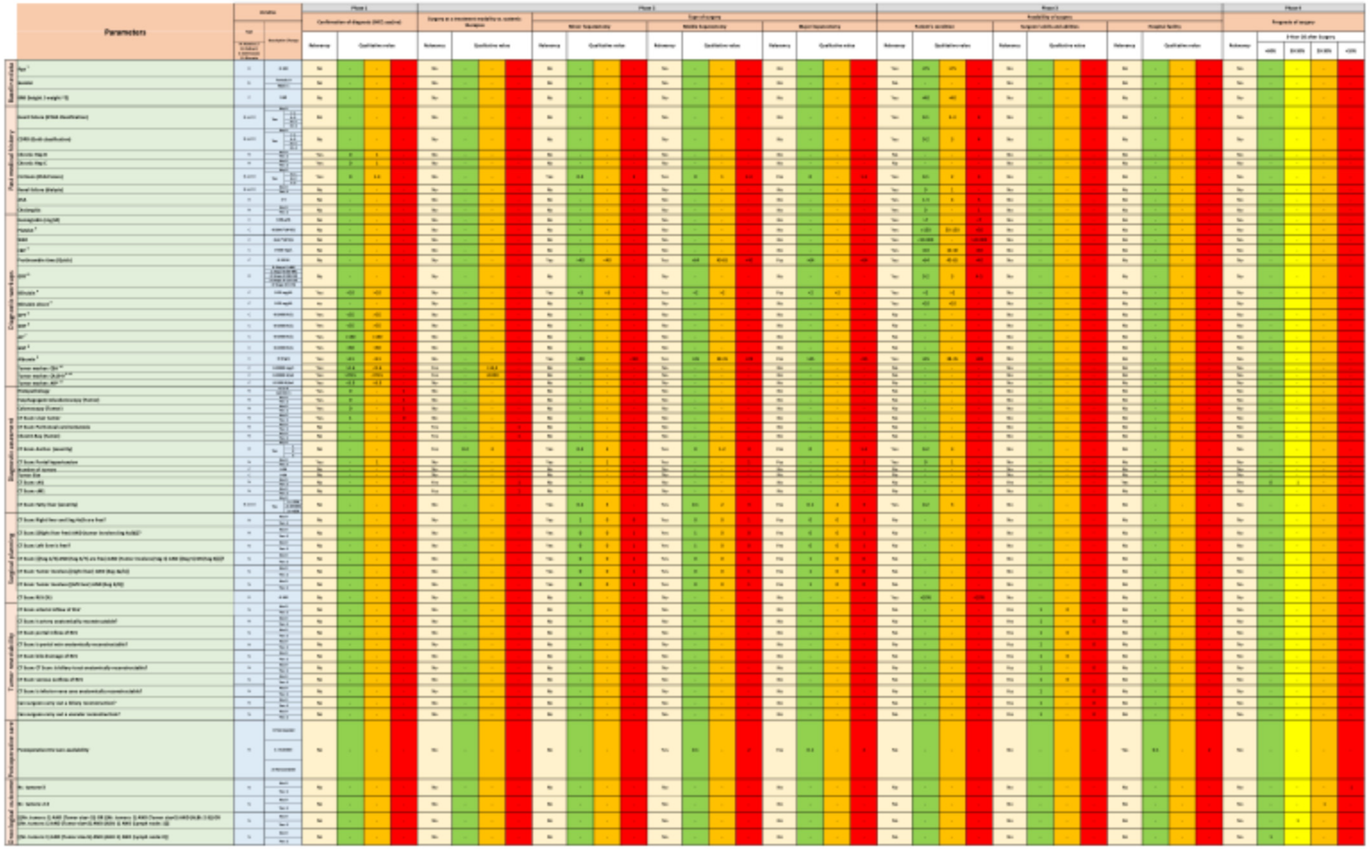

References

1. Vitale A, Spolverato G, Bagante F, Gani F, Popescu I, Marques HP, et al. A multi-institutional analysis of elderly patients undergoing a liver resection for intrahepatic cholangiocarcinoma. J Surg Oncol. 2016;113(4):420-6.

2. Riediger CE, Löck S, Frohneberg L, Hoffmann R, Kahlert C, Weitz J. Oncological liver resection in elderly - A retrospective comparative study. Int J Surg. 2022;104:106729.

3. Zou Y, Xu X, Wu T, Chen Q, Li Z, Yang Z, et al. Sex disparity in clinical characteristics and long-term prognosis after liver resection for patients with intrahepatic cholangiocarcinoma: A propensity score matching analysis. Heliyon. 2024;10(9):e29910.

4. Merath K, Mehta R, Hyer JM, Bagante F, Sahara K, Alexandrescu S, et al. Impact of body mass index on tumor recurrence among patients undergoing curative-intent resection of intrahepatic cholangiocarcinoma- a multi-institutional international analysis. Eur J Surg Oncol. 2019;45(6):1084-91.

5. Yugawa K, Itoh S, Iseda N, Kurihara T, Kitamura Y, Toshima T, et al. Obesity is a risk factor for intrahepatic cholangiocarcinoma progression associated with alterations of metabolic activity and immune status. Sci Rep. 2021;11(1):5845.

6. Zhao L, Wang J, Kong J, Zheng X, Yu X. The impact of body mass index on short-term and long-term surgical outcomes of laparoscopic hepatectomy in liver carcinoma patients: a retrospective study. World J Surg Oncol. 2022;20(1):150.

7. Lopez-Lopez V, Morise Z, Gomez Gavara C, Gero D, Abu Hilal M, Goh BK, et al. Global Outcomes Benchmarks in Laparoscopic Liver Surgery for Segments 7 and 8: International Multicenter Analysis. J Am Coll Surg. 2024;239(4):375-86.

8. Jehan FS, Ganguli S, Hase NE, Seth A, Kwon Y, Hemming AW, et al. Does the Surgical Approach Affect the Incidence of Post-Hepatectomy Liver Failure in Cirrhotic Patients? An Analysis of the NSQIP Database. Am Surg. 2024;90(11):2901-6.

9. Wang Q, Li J, Lei Z, Wu D, Si A, Wang K, et al. Prognosis of Intrahepatic Cholangiocarcinomas with HBV Infection is Better than Those with Hepatolithiasis After R0 Liver Resection: A Propensity Score Matching Analysis. Ann Surg Oncol. 2017;24(6):1579-87.

10. Jesper D, Heyn SG, Schellhaas B, Pfeifer L, Goertz RS, Zopf S, et al. Effects of liver cirrhosis and patient condition on clinical outcomes in intrahepatic cholangiocarcinoma: a retrospective analysis of 156 cases in a single center. Eur J Gastroenterol Hepatol. 2018;30(5):552-6.

11. Cipriani F, Aldrighetti L, Ratti F, Wu AGR, Kabir T, Scatton O, et al. Impact of Liver Cirrhosis, Severity of Cirrhosis, and Portal Hypertension on the Difficulty and Outcomes of Laparoscopic and Robotic Major Liver Resections for Primary Liver Malignancies. Ann Surg Oncol. 2024;31(1):97-114.

12. Bagante F, Alaimo L, Tsilimigras D, Dalbeni A, Ejaz A, Ruzzenente A, et al. Kidney Disease: Improving Global Outcomes Classification of Chronic Kidney Disease and Short-Term Outcomes of Patients Undergoing Liver Resection. J Am Coll Surg. 2022;234(5):827-39.

13. Yoshikawa T, Nomi T, Hokuto D, Kamitani N, Matsuo Y, Sho M. Outcomes in Patients with Chronic Kidney Disease After Liver Resection for Hepatocellular Carcinoma. World J Surg. 2021;45(2):598-606.

14. van Keulen AM, Olthof PB, Buettner S, Bednarsch J, Verheij J, Erdmann JI, et al. The Influence of Hepatic Steatosis and Fibrosis on Postoperative Outcomes After Major Liver Resection of Perihilar Cholangiocarcinoma. Ann Surg Oncol. 2024;31(1):133-41.

15. Badawy A, Kaido T, Hammad A, Yagi S, Kamo N, Yoshizawa A, et al. The Impact of Preoperative Hemoglobin Level on the Short-Term Outcomes After Living Donor Liver Transplantation. World J Surg. 2018;42(12):4081-9.

16. Giehl-Brown E, Geipel E, Löck S, Dehlke K, Schweipert J, Weitz J, et al. Transfusions of packed red blood cells in surgery for liver cancer: predictor of impaired overall survival but not recurrence-free survival - impact of blood transfusions in liver surgery. J Gastrointest Surg. 2024;28(4):402-11.

17. Chatzipanagiotou OP, Tsilimigras DI, Catalano G, Ruzzenente A, Aldrighetti L, Weiss M, et al. Preoperative platelet count as an independent predictor of long-term outcomes among patients undergoing resection for intrahepatic cholangiocarcinoma. J Surg Oncol. 2024.

18. Chen Q, Dai Z, Yin D, Yang LX, Wang Z, Xiao YS, et al. Negative impact of preoperative platelet-lymphocyte ratio on outcome after hepatic resection for intrahepatic cholangiocarcinoma. Medicine (Baltimore). 2015;94(13):e574.

19. Margonis GA, Amini N, Buettner S, Besharati S, Kim Y, Sobhani F, et al. Impact of early postoperative platelet count on volumetric liver gain and perioperative outcomes after major liver resection. Br J Surg. 2016;103(7):899-907.

20. Ito T, Shinkawa H, Takemura S, Tanaka S, Nishioka T, Miyazaki T, et al. Impact of the Preoperative C-reactive Protein to Albumin Ratio on the Long-Term Outcomes of Hepatic Resection for Intrahepatic Cholangiocarcinoma. Asian Pac J Cancer Prev. 2020;21(8):2373-9.

21. Nakao Y, Yamashita YI, Arima K, Miyata T, Itoyama R, Yusa T, et al. Clinical Usefulness of Perioperative C-reactive Protein/Albumin Ratio in Patients With Intrahepatic Cholangiocarcinoma: A Retrospective Single Institutional Study. Anticancer Res. 2019;39(5):2641-6.

22. Lin ZY, Liang ZX, Zhuang PL, Chen JW, Cao Y, Yan LX, et al. Intrahepatic cholangiocarcinoma prognostic determination using pre-operative serum C-reactive protein levels. BMC Cancer. 2016;16(1):792.

23. Yokoyama Y, Ebata T, Igami T, Sugawara G, Ando M, Nagino M. Predictive power of prothrombin time and serum total bilirubin for postoperative mortality after major hepatectomy with extrahepatic bile duct resection. Surgery. 2014;155(3):504-11.

24. Wang HS, Ge XX, Li QP, Nie JJ, Miao L. Clinical Significance of Prothrombin Time in Cholangiocarcinoma Patients with Surgeries. Can J Gastroenterol Hepatol. 2019;2019:3413969.

25. Iwasaki Y, Sawada T, Mori S, Iso Y, Katoh M, Rokkaku K, et al. Estimating glomerular filtration rate preoperatively for patients undergoing hepatectomy. World J Gastroenterol. 2009;15(18):2252-7.

26. Munir MM, Endo Y, Lima HA, Alaimo L, Moazzam Z, Shaikh C, et al. Albumin-Bilirubin Grade and Tumor Burden Score Predict Outcomes Among Patients with Intrahepatic Cholangiocarcinoma After Hepatic Resection: a Multi-Institutional Analysis. J Gastrointest Surg. 2023;27(3):544-54.

27. Kirkland M, Verhoeff K, Jogiat U, Mocanu V, Shapiro AMJ, Anderson B, et al. Persistent hyperbilirubinemia following preoperative biliary stenting in patients undergoing anatomic hepatectomy predicts serious complications. Surg Endosc. 2024;38(8):4287-95.

28. Shen J, Wen T, Li C, Yan L, Li B, Yang J. The Prognostic Prediction Role of Preoperative Serum Albumin Level in Patients with Intahepatic Cholangiocarcinoma Following Hepatectomy. Dig Dis. 2018;36(4):306-13.

29. Tsilimigras DI, Hyer JM, Moris D, Sahara K, Bagante F, Guglielmi A, et al. Prognostic utility of albumin-bilirubin grade for short- and long-term outcomes following hepatic resection for intrahepatic cholangiocarcinoma: A multi-institutional analysis of 706 patients. J Surg Oncol. 2019;120(2):206-13.

30. Zhang F, Lu S, Tian M, Hu K, Chen R, Zhang B, et al. Albumin-to-Alkaline Phosphatase Ratio is an Independent Prognostic Indicator in Combined Hepatocellular and Cholangiocarcinoma. J Cancer. 2020;11(17):5177-86.

31. Moro A, Mehta R, Sahara K, Tsilimigras DI, Paredes AZ, Farooq A, et al. The Impact of Preoperative CA19-9 and CEA on Outcomes of Patients with Intrahepatic Cholangiocarcinoma. Ann Surg Oncol. 2020;27(8):2888-901.

32. Fu J, Zheng L, Tang S, Lin K, Zheng S, Bi X, et al. Tumor burden score and carcinoembryonic antigen predict outcomes in patients with intrahepatic cholangiocarcinoma following liver resection: a multi-institutional analysis. BMC Cancer. 2024;24(1):358.

33. Fu J, Chen Q, Yu Y, You W, Ding Z, Gao Y, et al. Impact of portal hypertension on short- and long-term outcomes after liver resection for intrahepatic cholangiocarcinoma: A propensity score matching analysis. Cancer Med. 2021;10(20):6985-97.

34. Jansson H, Villard C, Nooijen LE, Ghorbani P, Erdmann JI, Sparrelid E. Prognostic influence of multiple hepatic lesions in resectable intrahepatic cholangiocarcinoma: A systematic review and meta-analysis. Eur J Surg Oncol. 2023;49(4):688-99.

35. Buettner S, Ten Cate DWG, Bagante F, Alexandrescu S, Marques HP, Lamelas J, et al. Survival after Resection of Multiple Tumor Foci of Intrahepatic Cholangiocarcinoma. J Gastrointest Surg. 2019;23(11):2239-46.

36. Nassar A, Tzedakis S, Sindayigaya R, Hobeika C, Marchese U, Veziant J, et al. Factors of Early Recurrence After Resection for Intrahepatic Cholangiocarcinoma. World J Surg. 2022;46(10):2459-67.

37. Kong J, Cao Y, Chai J, Liu X, Lin C, Wang J, et al. Effect of Tumor Size on Long-Term Survival After Resection for Solitary Intrahepatic Cholangiocarcinoma. Front Oncol. 2020;10:559911.

38. Kanu EN, Rhodin KE, Masoud SJ, Eckhoff AM, Bartholomew AJ, Howell TC, et al. Tumor size and survival in intrahepatic cholangiocarcinoma treated with surgical resection or ablation. J Surg Oncol. 2023;128(8):1329-39.

39. Jolissaint JS, Soares KC, Seier KP, Kundra R, Gönen M, Shin PJ, et al. Intrahepatic Cholangiocarcinoma with Lymph Node Metastasis: Treatment-Related Outcomes and the Role of Tumor Genomics in Patient Selection. Clin Cancer Res. 2021;27(14):4101-8.

40. Zhang XF, Xue F, Dong DH, Weiss M, Popescu I, Marques HP, et al. Number and Station of Lymph Node Metastasis After Curative-intent Resection of Intrahepatic Cholangiocarcinoma Impact Prognosis. Ann Surg. 2021;274(6):e1187-e95.

41. Kim SH, Han DH, Choi GH, Choi JS, Kim KS. Prognostic impact of the metastatic lymph node number in intrahepatic cholangiocarcinoma. Surgery. 2022;172(1):177-83.

42. Orimo T, Kamiyama T, Mitsuhashi T, Kamachi H, Yokoo H, Wakayama K, et al. Impact of tumor localization on the outcomes of surgery for an intrahepatic cholangiocarcinoma. Journal of Gastroenterology. 2018;53(11):1206-15.

43. Li H, Liu R, Li J, Li J, Wu H, Wang G, et al. Tumor location influences perioperative and oncologic outcomes in solitary intrahepatic cholangiocarcinoma following curative resection: a multi-center analysis. HPB (Oxford). 2022;24(9):1543-50.

44. Wang C, Ciren P, Danzeng A, Li Y, Zeng CL, Zhang ZW, et al. Anatomical Resection Improved the Outcome of Intrahepatic Cholangiocarcinoma: A Propensity Score Matching Analysis of a Retrospective Cohort. J Oncol. 2022;2022:4446243.

45. Bednarsch J, Czigany Z, Lurje I, Amygdalos I, Strnad P, Halm P, et al. Insufficient future liver remnant and preoperative cholangitis predict perioperative outcome in perihilar cholangiocarcinoma. HPB. 2021;23(1):99-108.

46. Reames BN, Ejaz A, Koerkamp BG, Alexandrescu S, Marques HP, Aldrighetti L, et al. Impact of major vascular resection on outcomes and survival in patients with intrahepatic cholangiocarcinoma: A multi-institutional analysis. J Surg Oncol. 2017;116(2):133-9.

47. Conci S, Viganò L, Ercolani G, Gonzalez E, Ruzzenente A, Isa G, et al. Outcomes of vascular resection associated with curative intent hepatectomy for intrahepatic cholangiocarcinoma. European Journal of Surgical Oncology. 2020;46(9):1727-33.

48. Lafaro KJ, Cosgrove D, Geschwind J-FH, Kamel I, Herman JM, Pawlik TM. Multidisciplinary Care of Patients with Intrahepatic Cholangiocarcinoma: Updates in Management. Gastroenterology Research and Practice. 2015;2015(1):860861.
